# Supplementary material for: Benefits of integrated screening and vaccination for infection control
Source: PLoS One. 2022 Apr 21;17(4):e0267388. doi: 10.1371/journal.pone.0267388 (PMC9023060; doi:10.1371/journal.pone.0267388)
Supplement: S1 Appendix — (PDF) [file pone.0267388.s001.pdf]

S1 Appendix: Supplementary Online Content

Marie Jeanne Rabil, Sait Tunc, Douglas R. Bish, Ebru K. Bish

Model Description

We developed an extended SEIR (Susceptible, Exposed, Infectious, Removed) framework to model the COVID-19 spread in a population composed of two homogeneous groups (faculty and students) under protective and preventative interventions including screening, isolation, and vaccination. The model tracks the population of at-risk individuals while they transition through different health states, or *compartments*, where each transition is modeled probabilistically and the overall flow is governed by a series of difference equations. Our model provides an extension to the conventional SIR frameworks with mass vaccination by generalizing the SEIR compartmental model of [1], which was introduced to study the epidemiology and natural history of COVID-19 infection. In particular, we expanded the compartmental model in [1] in the following ways, which are also illustrated in the flow chart given in Fig A:

- Modeling on-campus vaccination, which is performed concurrently with isolation and screening.
- Modeling the probabilistic time to immunity after vaccination.
- Distinguishing between vaccinated (but not fully immune) and unvaccinated individuals in the active transmission and screening pool.
- Recognizing the potential "immunity unknowing" by differentiating between the individuals with a detected infection (through either having symptoms or having a positive

PCR test) or vaccination and the ones that got recovered from the infection unknowingly because of the absence of symptoms or a positive PCR test.

- Modeling the option to screen the vaccinated individuals.
- Modeling population-based disparities in disease spread, hospitalization, and mortality rates, by modeling two groups, i.e., students and faculty, and their interactions.
- Modeling imperfect compliance with vaccination and screening guidelines.
- Modeling imperfect vaccine efficacy.
- Modeling the hospitalization rate for symptomatic cases.

Fig A: Flow diagram of the extended SEIR model

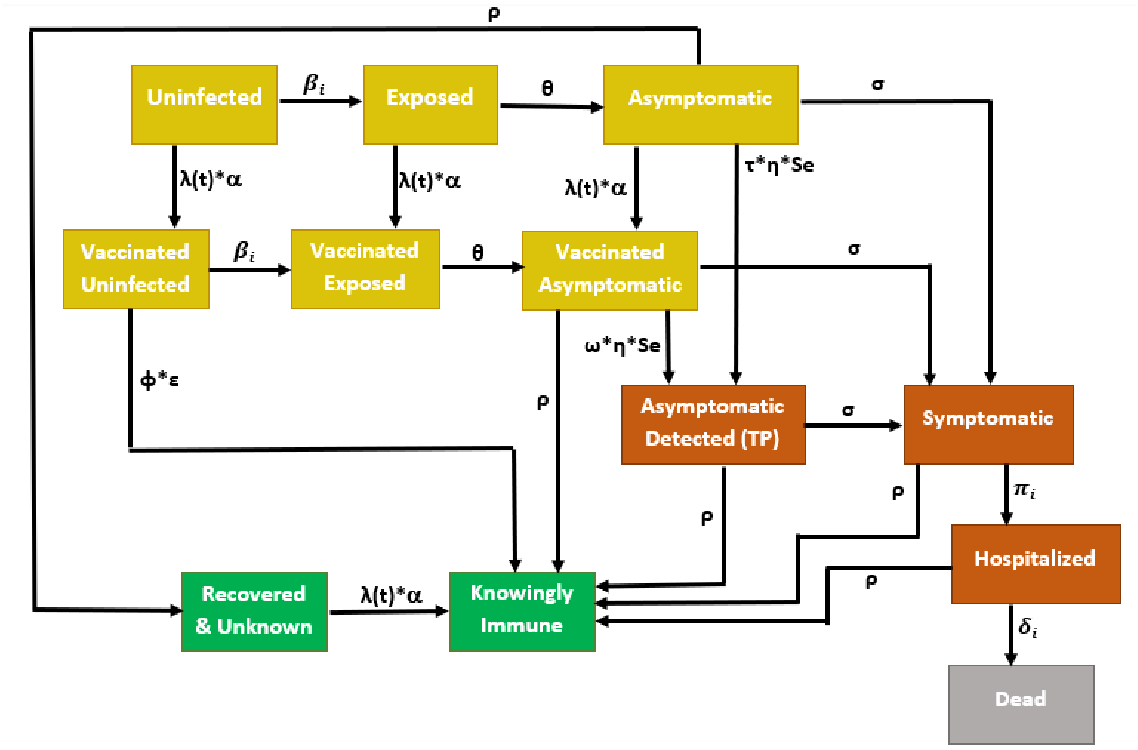

Compartments

We defined a total of 15 model compartments for each population subgroup i, where $i \in \{students, faculty\}$, and kept the inherited compartments from [1] with their original

names to highlight the similarities and differences between the two models.

Unvaccinated transmission and screening pool. All individuals in this pool can be vaccinated. This pool can also be tested for infection.

- U_i: Uninfected, unvaccinated, susceptible.
- E_i: Exposed, unvaccinated, asymptomatic, non-infectious.
- A_i: Asymptomatic, unvaccinated, infected.

Vaccinated transmission and screening pool. All individuals in this pool are assumed to be vaccinated but not yet fully immune. Depending on the screening strategy, these individuals can be tested for infection. Along with the *Unvaccinated transmission and screening pool*, this pool contains all individuals who can transmit an infection or can get infected.

- VU_i: Vaccinated, uninfected, susceptible.
- VE_i: Vaccinated, exposed, asymptomatic, non-infectious.
- VA_i: Vaccinated, asymptomatic, infected.

Unknowingly recovered screening pool. All the individuals in this pool are assumed to be recovered from infection unknowingly. Together with *Unvaccinated transmission and screening pool* and *Vaccinated transmission and screening pool*, these pools are assumed to include all individuals that can be tested for infection.

- RU_i: Recovered & Unknown, i.e., those who were infected and recovered, but did not know of their infection.

Isolation pool. All individuals in this pool are assumed to be isolated from each other as well as from the other pools. No transmission takes places within this pool.

- FP_i: False-Positive result, uninfected.
- $FPVU_i$: False-Positive, Vaccinated & Uninfected.

- $FPRU_i$: False-Positive, Recovered & Unknown.
- TP_i (Asymptomatic Detected): True Positive result, asymptomatic, infected.
- S_i: Symptomatic, detected.
- H_i: Hospitalized.

Removed pool. Individuals in this pool are assumed to be immune for at least 80 days (the duration of the semester). Accordingly, we assume that no transmission, screening or vaccination takes place for individuals in this pool.

- D_i: Dead.
- KI_i: Knowingly immune, i.e., those who were either infected and recovered, and knew of their infection, or vaccinated and built immunity.

A summary of the compartmental flow assumptions is given in Table A.

Table A: Summary of compartmental flow assumptions

**Compartment**	**In screening pool?**	**In vaccination pool?**	**In isolation pool?**
(U_i)–Uninfected	Yes	Yes	No
(E_i)–Exposed	Yes	Yes	No
(A_i)–Asymptomatic	Yes	Yes	No
(RU_i)–Recovered & Unknown	Yes	Yes	No
(VU_i)–Vaccinated Uninfected	Depends	No	No
(VA_i)–Vaccinated Asymptomatic	Depends	No	No
(VE_i)–Vaccinated Exposed	Depends	No	No
(FP_i)–False-Positive	No	No	Yes
$(FPVU_i)$–False-Positive, Vaccinated & Uninfected	No	No	Yes
$(FPRU_i)$–False-Positive, Recovered & Unknown	No	No	Yes
(TP_i)–True-Positive, Asymptomatic Detected	No	No	Yes
(S_i)–Symptomatic	No	No	Yes
(H_i)–Hospitalized	No	No	Yes
(KI_i)–Knowingly Immune	No	No	No
(D_i)–Dead	No	No	No

Model Parameters

Subscript $i, j \in \{\text{students}, \text{faculty}\}$ denotes the subgroup of the population. Parameters without these subscripts are the same for both subgroups.

$\beta_{j,i}$:	rate at which infected subjects in group j contact subjects from group i and infect them
τ:	rate at which unvaccinated subjects in the screening pool are screened for infection
θ:	rate at which exposed subjects advance to the Asymptomatic & infectious compartment
δ_i:	rate at which subjects of group i in the Hospitalized compartment die
π_i:	rate at which subjects of group i in the Symptomatic compartment get hospitalized
ρ:	rate at which infected subjects recover from disease
σ:	rate of symptom onset for infected subjects
μ:	rate at which false-positives are returned to the Uninfected compartment
$sens$:	sensitivity of the screening test
$spec$:	specificity of the screening test
I(t):	an indicator function which assumes a value 1 if an exogenous shock takes place in cycle t; 0 Otherwise
X:	number of imported infections to group i in a given exogenous shock
ω:	rate at which vaccinated subjects in the screening pool are screened for infection
$\lambda(t)$:	rate at which eligible subjects are vaccinated
ϕ:	rate at which vaccinated subjects advance to the "Immune & known" compartment,
α:	vaccine compliance rate
η:	screening compliance rate
ϵ:	vaccine efficacy

The model uses a cycle time of 8 hours. Screening rates τ and ω are assumed to be static, whereas the vaccination rate $\lambda(t)$ is time-dependent so that a constant number of vaccines are administered per day. Certain parameter values are varied in the analysis to simulate

different strategies or scenarios, e.g., vaccination rate, screening frequency, etc.

Governing Equations

The following defines the governing equations for the model depicted in Fig A, where $i, j \in \{\text{students}, \text{faculty}\}$ and $Z_i(t) := U_i(t) + VU_i(t) + E_i(t) + VE_i(t) + A_i(t) + VA_i(t) + RU_i(t) + KI_i(t)$.

$$U_i(t+1) = U_i(t) \cdot \left[1 - \sum_j \left[\beta_{j,i} \cdot \frac{A_j(t) + VA_j(t)}{Z_j(t)}\right] - \lambda(t) \cdot \alpha\right] - U_i(t-1) \cdot \tau \cdot \eta \cdot (1 - spec) + \mu \cdot FP_i(t) - X \cdot I(t+1)$$

$$E_i(t+1) = E_i(t) \cdot [1 - \theta - \lambda(t) \cdot \alpha] + \sum_j \left[\beta_{j,i} \cdot \frac{U_i(t) \cdot [A_j(t) + VA_j(t)]}{Z_j(t)}\right] + X \cdot I(t+1)$$

$$A_i(t+1) = A_i(t) \cdot [1 - \sigma - \rho - \lambda(t) \cdot \alpha] - A_i(t-1) \cdot \tau \cdot \eta \cdot sens + E_i(t) \cdot \theta$$

$$FP_i(t+1) = FP_i(t) \cdot [1 - \mu] + U_i(t-1) \cdot \tau \cdot \eta \cdot (1 - spec)$$

$$TP_i(t+1) = TP_i(t) \cdot [1 - \sigma - \rho] + A_i(t-1) \cdot \tau \cdot \eta \cdot sens + VA_i(t-1) \cdot \omega \cdot \eta \cdot sens$$

$$S_i(t+1) = S_i(t) \cdot [1 - \rho - \pi_i] + \sigma \cdot [TP_i(t) + A_i(t) + VA_i(t)]$$

$$H_i(t+1) = H_i(t) \cdot [1 - \rho - \delta_i] + \pi_i \cdot S_i(t)$$

$$KI_i(t+1) = KI_i(t) + \rho \cdot [TP_i(t) + S_i(t) + H_i(t) + VA_i(t)] + \lambda(t) \cdot \alpha \cdot RU_i(t) + \phi \cdot \epsilon \cdot VU_i(t)$$

$$RU_i(t+1) = RU_i(t) \cdot (1 - \lambda(t) \cdot \alpha) + \rho \cdot A_i(t) - RU_i(t-1) \cdot \tau \cdot \eta \cdot (1 - spec) + \mu \cdot FPRU_i(t)$$

$$D_i(t+1) = D_i(t) + \delta_i \cdot H_i(t)$$

$$FPVU_i(t+1) = FPVU_i(t) \cdot [1 - \mu] + VU_i(t-1) \cdot \omega \cdot \eta \cdot (1 - spec)$$

$$FPRU_i(t+1) = FPRU_i(t) \cdot [1 - \mu] + RU_i(t-1) \cdot \tau \cdot \eta \cdot (1 - spec)$$

$$VU_i(t+1) = VU_i(t) \cdot \left[1 - \phi \cdot \epsilon - \sum_j \left[\beta_{j,i} \cdot \frac{VA_j(t) + A_j(t)}{Z_j(t)}\right]\right] + \lambda(t) \cdot \alpha \cdot U_i(t) - VU_i(t-1) \cdot \omega \cdot \eta \cdot (1 - spec) + \mu \cdot FPVU_i(t)$$

$$VE_i(t+1) = VE_i(t) \cdot (1 - \theta) + \sum_j \left[\beta_{j,i} \cdot \frac{VU_i(t)[VA_j(t) + A_j(t)]}{Z_j(t)}\right] + \lambda(t) \cdot \alpha \cdot E_i(t)$$

$$VA_i(t+1) = VA_i(t)\cdot(1-\sigma-\rho) + \lambda(t)\cdot\alpha\cdot A_i(t) - VA_i(t-1)\cdot\omega\cdot\eta\cdot sens + VE_i(t)\cdot\theta$$

$$N = \sum_i \Big[U_i + E_i + A_i + S_i + TP_i + FP_i + KI_i + H_i + RU_i + D_i + FPVU_i + FPRU_i + VU_i + VA_i + VE_i\Big]$$

Initial Conditions

- $KI_i(0) = \begin{cases} \{1500, 3000, 4500\}, & i = students \\ \{136, 273, 409\}, & i = faculty \end{cases}$

- $A_i(0) = \begin{cases} 10, & i = students \\ 1, & i = faculty \end{cases}$

- $U_i(0) = \begin{cases} 5000 - A_i(0) - KI_i(0), & i = students \\ 455 - A_i(0) - KI_i(0), & i = faculty \end{cases}$

All other compartments are initially empty. Accordingly, $N = 5000 + 455 = 5,455$.

Estimating Key Rate Parameters

The following parameters are inherited from [1]:

- σ, rate of symptom onset for infected individuals: σ is estimated by solving $\sigma/(\sigma+\rho) = 30\%$, where 30% [2] is the probability of getting symptoms after an infection and ρ is the rate of recovery. Since the mean time to recovery is 14 days and each day is composed of 3 eight-hour cycles, we have $\rho = 1/(3\cdot 14)$, leading to $\sigma = 0.0102$.

- τ, rate at which unvaccinated individuals in the screening pool are screened for infection: It is given by $\tau = \frac{1}{3\cdot f}$, where f is the screening frequency, which can be *daily, every 2 days, every 3 days, every 7 days or every 14 days.*

- θ, rate at which exposed subjects advance to the Asymptomatic & infectious compartment: It is given by $\theta = \frac{1}{3 \cdot 3} = 0.111$ since the mean latent period is 3 days and each day is composed of 3 eight-hour cycles.

In addition, we use the following estimates.

- $\beta_{j,i}$, rate at which infected subjects in group j contact subjects from group i and infect them, where $i, j \in \{\text{students}, \text{faculty}\}$. It varies based on the gravity of transmission (best, base or worst case scenario) which is represented in terms of reproduction number $R_{T(j,i)}$. For notational simplicity, we denote students with 's' and faculty with 'f' in the remainder of the paragraph. Based on the type of dynamics, we assume that $R_{T(s,s)} = 5.4$, $R_{T(s,f)} = 0.6$, $R_{T(f,f)} = 0.32$ and $R_{T(f,s)} = 2.88$. Thus, we get that the overall $R_{T(s)} = 6$ and $R_{T(f)} = 3.2$ assuming a 11:1 student to faculty ratio (based on [3]). As a result, the population reproduction number R_T for the base-case is $R_T = 0.09 \cdot R_{T(f)} + 0.91 \cdot R_{T(s)} = 5.75$. $\beta_{j,i}$ is estimated by solving $R_{T(j,i)} = \beta_{j,i}/(\sigma + \rho)$, which gives $\beta_{s,s} = 0.184$, $\beta_{s,f} = 0.0204$, $\beta_{f,f} = 0.0109$ and $\beta_{f,s} = 0.098$ for the base-case scenario.

  For the best-case and worst-case scenarios, we set R_T to 6.75 and 4.75, respectively. Accordingly, under these scenarios, $R_{T(s)}$ becomes 5 and 7 and $R_{T(f)}$ becomes 2.2 and 4.2, respectively. Therefore, we have $R_{T(s,s)} = 4.5$, $R_{T(s,f)} = 0.5$, $R_{T(f,f)} = 0.22$ and $R_{T(f,s)} = 1.98$ (i.e., $\beta_{s,s} = 0.153$, $\beta_{s,f} = 0.017$, $\beta_{f,f} = 0.00748$ and $\beta_{f,s} = 0.0673$) for the best-case scenario and $R_{T(s,s)} = 6.3$, $R_{T(s,f)} = 0.7$, $R_{T(f,f)} = 0.42$ and $R_{T(f,s)} = 3.78$ (i.e., $\beta_{s,s} = 0.214$, $\beta_{s,f} = 0.0238$, $\beta_{f,f} = 0.0143$ and $\beta_{f,s} = 0.128$) for the worst-case scenario.

- π_i, rate at which subjects in the *Symptomatic* compartment of group i get hospitalized, where $i \in \{students, faculty\}$. These rates are calculated based on the hospitalization rates of 1.4% and 8.4% for students and faculty, respectively [4,5]. The hospitalization rates are obtained by the current and cumulative rate of COVID-19 hospitalizations

per age group (with respect to the total population) [4] and the total percentage of infections per age group [5]. For each age group, the hospitalization rate (among the infected subjects) is the ratio of the corresponding rate of hospitalization to the percentage of infections. Assuming all university students to be in the 18-29 age group, we get a hospitalization rate of 1.4% for students. For the faculty, we consider that their age range covers multiple age groups (30-39, 40-49, 50-64, 65-74 and 75-84) [6]. The percentage of faculty in each of these five age groups are estimated as 17%, 33%, 37%, 10%, and 3%, respectively, with the corresponding hospitalization rates of 2.9%, 4.8%, 5.7%, 21.5% and 69.3%, respectively. Using a weighted average yields a faculty hospitalization rate of 8.4%. For $i \in \{\text{students}, \text{faculty}\}$, π_i is the solution to $[\sigma/(\rho+\sigma)] \cdot [\pi_i/(\rho+\pi_i)] = HospitalizationRate_i$, which gives $\pi_{student} = 0.001166$ and $\pi_{faculty} = 0.009263$.

- δ_i, rate at which subjects in the *Hospitalized* compartment of group i die. It is based on the fatality rate of 0.05% and 2% for students and faculty respectively [7,8]. The fatality rates are obtained using the current number of COVID-19 deaths per age group [7] and the current number of infections per age group [8]. For each age group, the fatality rate is the ratio of the corresponding number of deaths to the number of infections. Assuming all university students to be in the 18-29 age group, we get a fatality rate of 0.05% for students. Similar to the calculation of π_i, we consider five different age groups for the faculty with the corresponding fatality rates of 0.2%, 0.6%, 2%, 6%, and 14%, respectively. Using a weighted average yields a faculty fatality rate of 2%. For $i \in \{\text{students}, \text{faculty}\}$, δ_i is the solution to $[\sigma/(\rho+\sigma)] \cdot [\delta_i/(\rho+\delta_i)] \cdot [\pi_i/(\rho+\pi_i)] = FatalityRate_i$, which gives $\delta_{students} = 0.0008817$ and $\delta_{faculty} = 0.00744$.

- ϵ, vaccine efficacy: We consider only the 2-dose vaccines (Pfizer and Moderna) because they represent the majority of the vaccines administered in the US (58% and 38%, respectively), compared to around 4% of the 1-dose vaccine (Janssen) [9]. Accordingly,

the vaccine efficacy ϵ is calculated as the weighted average of Pfizer's efficacy of 95% [10] and Moderna's efficacy of 94.1%, with respective weights of 60% and 40% [11], that is, $\epsilon = 0.6 \cdot 95\% + 0.4 \cdot 94.1\% = 94.64\%$.

- η, compliance rate of screening: We use a baseline value of $\eta = 0.75$, and perform a sensitivity analysis at several levels of screening compliance using $\eta = \{0.5, 0.75, 1\}$.

- α, compliance rate of vaccination: We use a baseline value of $\alpha = 0.75$, and perform a sensitivity analysis at several levels of vaccination compliance using $\alpha = \{0.5, 0.75, 1\}$.

- ϕ, average time to full immunity after the second dose of the vaccine: The time to full immunity is estimated to be 5 or 6 weeks on average for Pfizer and Moderna, respectively [12]. Accordingly, using a weighted average, we obtain $\phi = \frac{1}{3\times 7\times(0.6\times 5+0.4\times 6)} = \frac{1}{3\times 7\times(5.4)} = 0.0089$.

- ω, rate at which vaccinated individuals in the screening pool are screened for infection: $\omega = \tau$ when vaccinated individuals are tested, $\omega = 0$ otherwise.

- $\lambda(t)$, rate at which eligible individuals are vaccinated: $\lambda(t)$ is time-dependent and updated dynamically so that the number of vaccines administered per time period (8 hours) is fixed. Initially, we set $\lambda(t+1) = \frac{VPP}{\sum_i [U_i(t)+E_i(t)+A_i(t)+RU_i(t)]}$ where VPP represents the number of vaccines per time period. In order to be able to vaccinate most of the students by the end of the semester, vaccine needs to be administered at a minimum of 60 vaccines per day. Accordingly, $VPP = \frac{60}{3} = 20$ is used in the base case, and a sensitivity analysis is performed over this value.

Fig A presents a flow diagram of the extended SEIR model. For clarity, Fig A does not include the false-positive compartments. Furthermore, for the compartments separately defined for students and faculty, only one compartment is shown in the figure.

(In the following tables, numbers were rounded to the nearest 10 when appropriate)

Table B: Total number of infections and peak number of hospitalizations over the 80-day semester for all strategies, implemented at various screening frequencies and daily vaccination rates, in the base-case scenario, for 75% compliance ($\eta = \alpha = 75\%$), 30% initial faculty vaccine coverage ($L_f = 30\%$), and various levels of initial student vaccine coverage (L_s).

		L_s : 30%, L_f : 30%		L_s : 60%, L_f : 30%		L_s : 90%, L_f : 30%	
Strategy	Test Frequency	Total number of infections	Peak number of hospitalizations	Total number of infections	Peak number of hospitalizations	Total number of infections	Peak number of hospitalizations
**120 vaccines/day**							
$V - S_F$	every 14d	2,420	7	400	1	9	0
	every 7d	2,020	6	320	1	8	0
	every 3 d	1,120	4	200	1	6	0
	every 2d	700	3	140	1	5	0
	every 1d	280	2	70	1	3	0
$V - S_P$	every 14d	2,560	8	450	1	9	0
	every 7d	2,350	7	390	1	8	0
	every 3d	1,840	5	300	1	7	0
	every 2d	1,530	4	240	1	6	0
	every 1d	1,020	3	160	1	4	0
$V - \overline{S}$	N/A	2,770	9	530	1	9	0
**60 vaccines/day**							
$V - S_F$	every 14d	3,010	13	1,030	5	33	0
	every 7d	2,750	12	790	4	29	0
	every 3 d	1,820	8	430	3	21	0
	every 2d	1,190	5	290	2	17	0
	every 1d	490	3	140	2	9	0
$V - S_P$	every 14d	3,080	14	1,130	5	36	0
	every 7d	2,940	13	970	4	33	0
	every 3d	2,540	10	710	3	26	0
	every 2d	2,220	9	570	3	23	0
	every 1d	1,600	6	380	2	16	0
$V - \overline{S}$	N/A	3,180	15	1,310	6	41	0
**30 vaccines/day**							
$V - S_F$	every 14d	3,230	14	1,410	6	90	1
	every 7d	3,030	13	1,160	5	70	1
	every 3 d	2,200	9	680	3	50	1
	every 2d	1,490	6	470	3	40	1
	every 1d	600	3	230	2	20	1
$V - S_P$	every 14d	3,260	14	1,480	7	100	1
	every 7d	3,130	13	1,320	6	90	1
	every 3d	2,680	10	980	4	70	1
	every 2d	2,260	8	790	4	50	1
	every 1d	1,300	5	490	3	40	1
$V - \overline{S}$	N/A	3,350	15	1,640	8	120	1
**No vaccination**							
$\overline{V} - \overline{S}$	N/A	3,510	16	1,900	9	320	3
$\overline{V} - S_F$	every 14d	3,430	15	1,720	7	250	2
	every 7d	3,280	14	1,480	6	200	2
	every 3d	2,560	10	910	4	130	2
	every 2d	1,820	7	630	3	100	2
	every 1d	720	3	300	2	60	2

Table C: Total number of infections and peak number of hospitalizations over the 80-day semester for all strategies, implemented at various screening frequencies and daily vaccination rates, in the base-case scenario, for 75% compliance ($\eta = \alpha = 75\%$), 30% initial student vaccine coverage ($L_s = 30\%$), and various levels of initial faculty vaccine coverage (L_f).

		L_s : 30%, L_f : 30%		L_s : 30%, L_f : 60%		L_s : 30%, L_f : 90%	
Strategy	Test Frequency	Total number of infections	Peak number of hospitalizations	Total number of infections	Peak number of hospitalizations	Total number of infections	Peak number of hospitalizations
**120 vaccines/day**							
$V - S_F$	every 14d	2,420	7	2,280	6	2,140	6
	every 7d	2,020	6	1,880	5	1,740	5
	every 3 d	1,120	4	1,030	3	950	3
	every 2d	700	3	640	3	590	2
	every 1d	280	2	260	2	240	2
$V - S_P$	every 14d	2,560	8	2,420	7	2,300	6
	every 7d	2,350	7	2,210	6	2,070	5
	every 3d	1,840	5	1,710	4	1,600	4
	every 2d	1,530	4	1,410	3	1,300	3
	every 1d	1,020	3	930	2	850	2
$V - \overline{S}$	N/A	2,770	9	2,650	8	2,540	7
**60 vaccines/day**							
$V - S_F$	every 14d	3,010	13	2,930	12	2,840	12
	every 7d	2,750	12	2,650	11	2,550	10
	every 3 d	1,820	8	1,720	7	1,620	7
	every 2d	1,190	5	1,110	5	1,040	5
	every 1d	490	3	460	3	430	3
$V - S_P$	every 14d	3,080	14	2,990	13	2,920	12
	every 7d	2,940	13	2,880	12	2,760	11
	every 3d	2,540	10	2,430	10	2,340	9
	every 2d	2,220	9	2,120	8	2,030	8
	every 1d	1,600	6	1,520	6	1,440	5
$V - \overline{S}$	N/A	3,180	15	3,110	13	3,040	13
**30 vaccines/day**							
$V - S_F$	every 14d	3,230	14	3,150	13	3,080	12
	every 7d	3,030	13	2,940	12	2,850	11
	every 3 d	2,200	9	2,100	8	2,000	8
	every 2d	1,490	6	1,410	5	1,330	5
	every 1d	600	3	580	3	550	3
$V - S_P$	every 14d	3,260	14	3,180	13	3,110	13
	every 7d	3,130	13	3,050	12	2,970	12
	every 3d	2,680	10	2,600	10	2,510	9
	every 2d	2,260	8	2,180	8	2,100	7
	every 1d	1,300	5	1,260	5	1,210	5
$V - \overline{S}$	N/A	3,350	15	3,280	14	3,210	13
**No vaccination**							
$\overline{V} - \overline{S}$	N/A	3,510	16	3,440	15	3,380	14
$\overline{V} - S_F$	every 14d	3,430	15	3,360	14	3,290	13
	every 7d	3,280	14	3,200	13	3,120	12
	every 3d	2,560	10	2,470	9	2,370	9
	every 2d	1,820	7	1,740	6	1650	6
	every 1d	720	3	700	3	660	3

Table D: Total number of infections and peak number of hospitalizations over the 80-day semester for various strategies, implemented at various screening frequencies and a daily vaccination rate of 60 vaccines/day, in the base-case scenario, for 60% initial campus-wide vaccine coverage ($L_s = L_f = 60\%$) and various screening (η) and vaccination (α) compliance rates.

		$\alpha = 50\%$		$\alpha = 75\%$		$\alpha = 95\%$	
Strategy	Test Frequency	Total number of infections	Peak number of hospitalizations	Total number of infections	Peak number of hospitalizations	Total number of infections	Peak number of hospitalizations
$\eta = 50\%$							
$V - S_F$	every 14d	1,280	6	1,010	4	790	3
	every 7d	1,110	5	850	4	660	2
	every 3 d	730	4	540	3	420	2
	every 2d	530	3	390	2	310	2
	every 1d	280	2	200	2	160	1
$V - S_P$	every 14d	1,340	6	1,080	4	850	3
	every 7d	1,230	6	970	4	770	3
	every 3d	980	5	760	3	600	2
	every 2d	820	4	630	3	500	2
	every 1d	560	3	430	2	340	2
$\eta = 75\%$							
$V - S_F$	every 14d	1,190	6	920	4	720	3
	every 7d	950	5	710	3	550	2
	every 3 d	530	3	390	2	310	2
	every 2d	370	3	270	2	220	2
	every 1d	190	2	140	2	110	1
$V - S_P$	every 14d	1,280	6	1,020	4	800	3
	every 7d	1,120	5	880	4	690	2
	every 3d	820	4	630	3	500	2
	every 2d	670	3	510	2	400	2
	every 1d	440	3	340	2	270	1
$\eta = 95\%$							
$V - S_F$	every 14d	1,120	5	860	4	670	2
	every 7d	830	4	620	3	490	2
	every 3 d	430	3	320	2	250	2
	every 2d	290	2	210	2	170	1
	every 1d	150	2	100	1	80	1
$V - S_P$	every 14d	1,240	6	980	4	770	3
	every 7d	1,050	5	810	3	640	2
	every 3d	730	4	560	3	440	2
	every 2d	580	3	450	2	350	2
	every 1d	380	2	300	2	230	1
**No screening**							
$V - \overline{S}$	N/A	1,450	7	1,200	5	950	3

Table E: Total number of infections and peak number of hospitalizations over the 80-day semester for all strategies, implemented at various screening frequencies and daily vaccination rates, in three severity scenarios, for 75% compliance ($\eta = \alpha = 75\%$) and 60% initial campus-wide vaccine coverage ($L_s = L_f = 60\%$).

		Base-case Scenario		Best-case Scenario		Worst-case Scenario	
Strategy	Test frequency	Total number of infections	Peak number of hospitalizations	Total number of infections	Peak number of hospitalizations	Total number of infections	Peak number of hospitalizations
**120 vaccines/day**							
$V - S_F$	every 14d	350	1	150	0	810	2
	every 7d	280	1	110	0	710	2
	every 3 d	170	1	70	0	500	2
	every 2d	130	1	50	0	390	1
	every 1d	70	1	30	0	220	1
$V - S_P$	every 14d	380	1	170	0	860	2
	every 7d	340	1	140	0	800	2
	every 3d	250	1	100	0	660	2
	every 2d	210	1	80	0	560	1
	every 1d	140	1	50	0	420	1
$V - \overline{S}$	N/A	450	1	200	1	950	2
**60 vaccines/day**							
$V - S_F$	every 14d	920	4	440	2	1,430	7
	every 7d	710	3	300	1	1,290	7
	every 3 d	390	2	150	1	940	5
	every 2d	270	2	100	1	730	5
	every 1d	140	2	50	1	410	4
$V - S_P$	every 14d	1,020	4	510	2	1,480	8
	every 7d	880	4	410	2	1,390	7
	every 3d	630	3	260	1	1,190	6
	every 2d	510	2	200	1	1,050	5
	every 1d	340	2	120	1	800	4
$V - \overline{S}$	N/A	1,200	5	660	2	1,550	8
**30 vaccines/day**							
$V - S_F$	every 14d	1,320	6	800	3	1,620	9
	every 7d	1,710	5	550	2	1,520	9
	every 3 d	640	3	260	1	1,220	8
	every 2d	440	3	170	1	1,000	7
	every 1d	220	2	80	1	610	5
$V - S_P$	every 14d	1,400	6	910	3	1,640	9
	every 7d	1,240	5	710	3	1,570	9
	every 3d	910	4	420	2	1,400	8
	every 2d	740	4	310	2	1,260	8
	every 1d	480	3	170	1	980	7
$V - \overline{S}$	N/A	1,560	7	1,170	4	1,690	10
**No vaccination**							
$\overline{V} - \overline{S}$	N/A	1,830	8	1,600	6	1,830	10
$\overline{V} - S_F$	every 14d	1,640	7	1,180	4	1,780	10
	every 7d	1,410	6	820	3	1,720	9
	every 3d	870	4	360	2	1,490	8
	every 2d	600	3	230	1	1,280	7
	every 1d	290	2	110	1	830	6

Table F: Total number of infections and peak number of hospitalizations over the 80-day semester for various strategies, implemented at various screening frequencies and a vaccination rate of 120 vaccines/day, in two severity scenarios for 75% compliance ($\eta = \alpha = 75\%$) and 30% initial campus-wide vaccine coverage ($L_s = L_f = 30\%$).

		Base-case Scenario		Worst-case Scenario	
Strategy	Test Frequency	Total number of infections	Peak number of hospitalizations	Total number of infections	Peak number of hospitalizations
**120 vaccines/day**					
$V - S_F$	every 14d	2,420	7	2,940	10
	every 7d	2,020	6	2,780	10
	every 3 d	1,120	4	2,260	8
	every 2d	700	3	1,770	7
	every 1d	280	2	900	5
$V - S_P$	every 14d	2,560	8	2,990	11
	every 7d	2,350	7	2,910	10
	every 3d	1,840	5	2,680	8
	every 2d	1,530	4	2,480	7
	every 1d	1,020	3	2,040	6
$V - \overline{S}$	N/A	2,770	9	3,060	11

Fig B: Peak number of hospitalizations versus total number of infections for 60% initial campus-wide vaccine coverage ($L_f = L_s = 60\%$) and 75% compliance ($\eta = \alpha = 75\%$) in three severity scenarios: (1) base-case, (2) worst-case, (3) best-case, (4) best-case figure enlarged, for total # of infections in [0,600]. All screening uses partial routine screening and N/A represents "no screening."

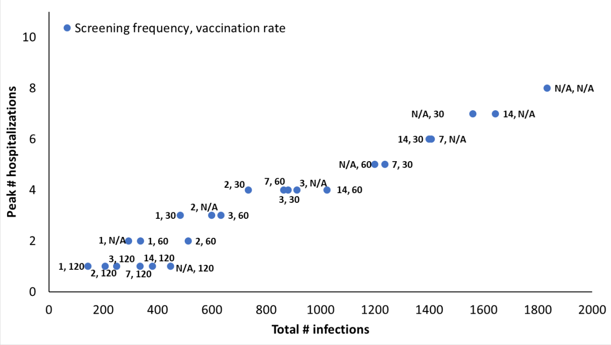

(1) Base-case scenario

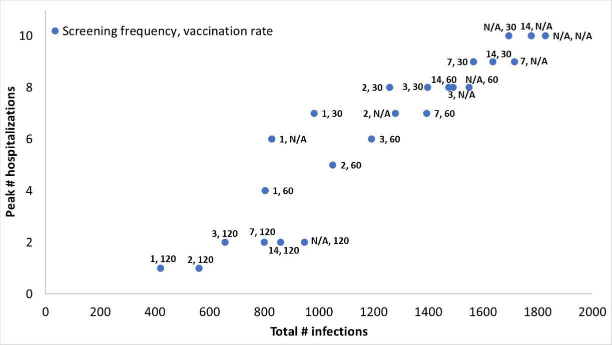

(2) Worst-case scenario

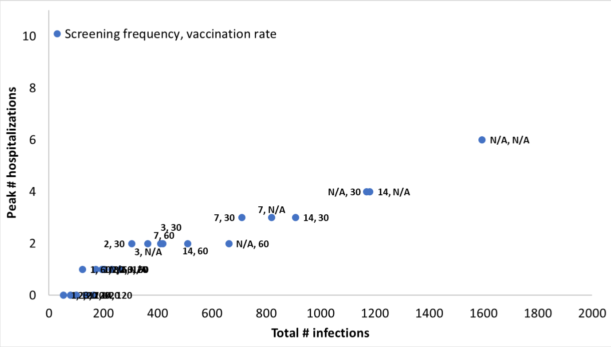

(3) Best-case scenario

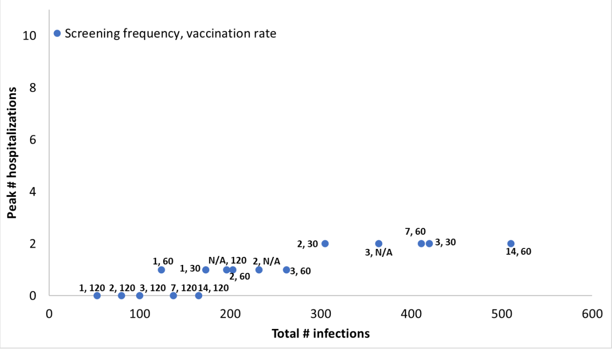

(4) Best-case scenario, enlarged

Fig C: Peak number of hospitalizations versus total number of infections in the base-case scenario, for $\eta = \alpha = 75\%$, 30% initial faculty vaccine coverage (L_f) and various levels of initial student vaccine coverage (L_s): (1) 30%, (2) 60%, (3) 90%, and (4) the figure in (c) enlarged, for total # of infections in [0,40]. All screening uses full routine screening and N/A represents "no screening."

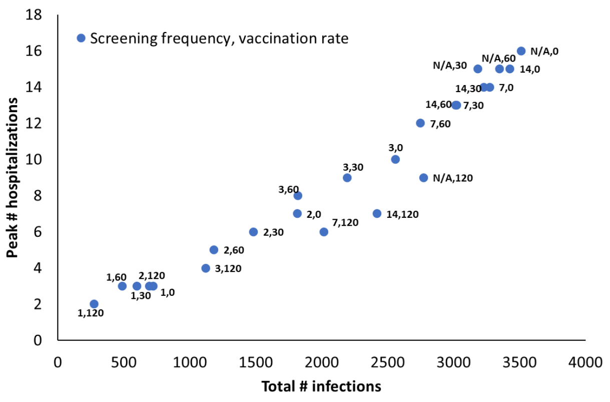

(1) $L_s = L_f = 30\%$

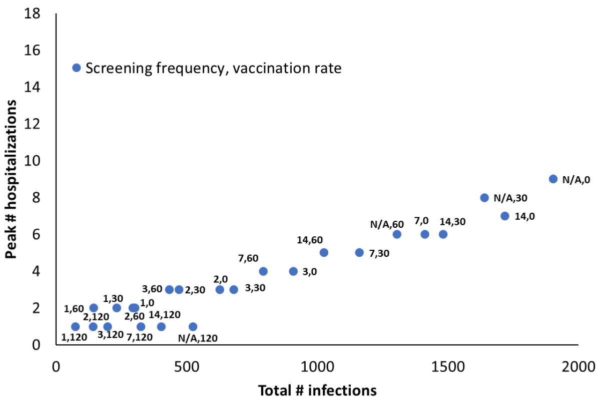

(2) $L_s = 60\%$ and $L_f = 30\%$

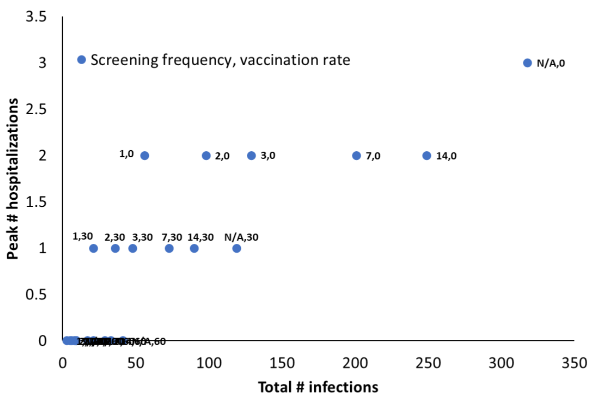

(3) $L_s = 90\%$ and $L_f = 30\%$

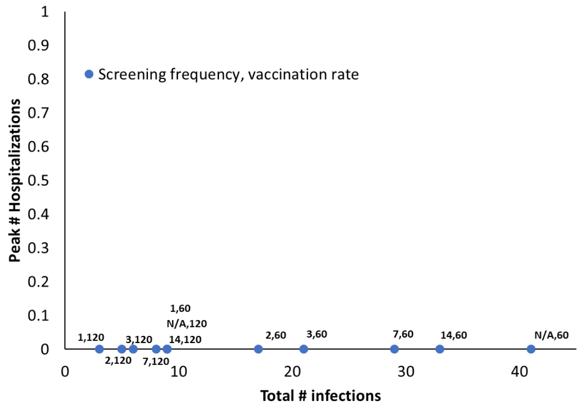

(4) $L_s = 90\%$ and $L_f = 30\%$, enlarged

Fig D: Total number of hospitalizations versus total number of infections in the base-case scenario with full screening for 60% initial campus-wide vaccine coverage ($L_s = L_f = 60\%$), 75% compliance ($\eta = \alpha = 75\%$), and various basic reproduction numbers and mean recovery times: (1) 5.75, 14 days, (2) 5.75, 5 days, (3) 3x5.75=17.25, 5 days. N/A represents "no screening."

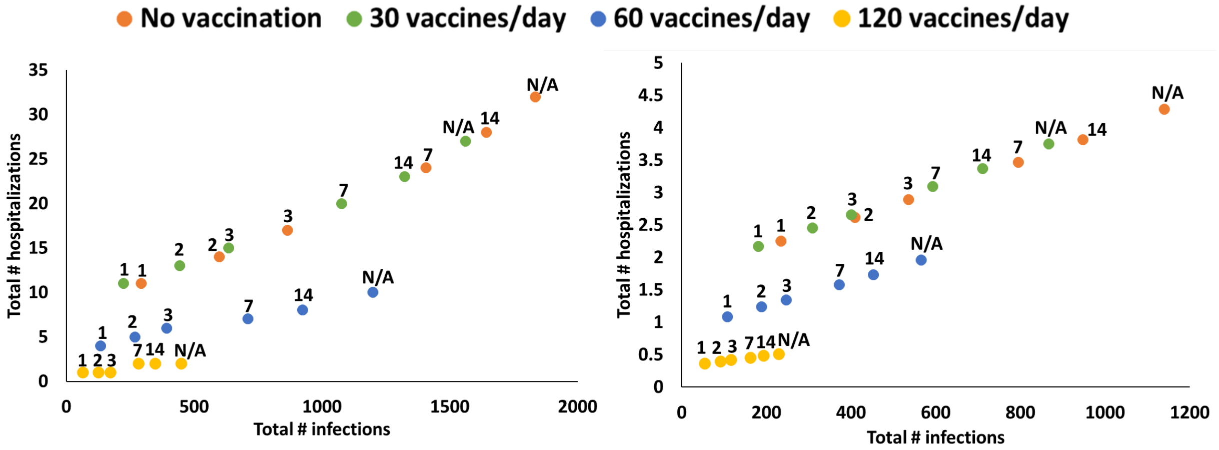

(1) $R_T = 5.75$ and mean recovery time of 14 days

(2) $R_T = 5.75$ and mean recovery time of 5 days

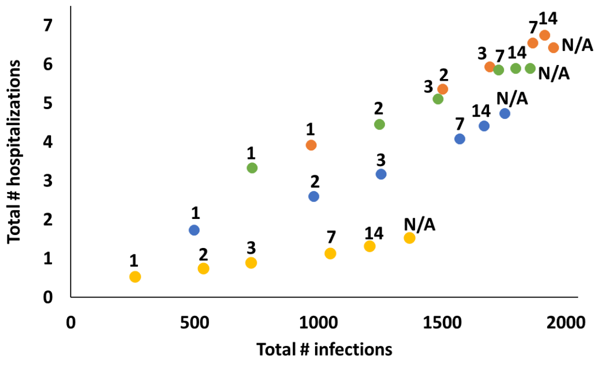

(3) $R_T = 3 \times 5.75$ and mean recovery time of 5 days

We note that the mean time to recovery for the Omicron variant is assumed to be 5 days [13] and the infectiousness (basic reproduction number) ratio Omicron:Delta is assumed to be 3:1 [14, 15].

Table G: Peak number, peak day, and number (students, faculty, overall) of infections, number of deaths, and peak and total number of hospitalizations (students, faculty, overall) over the 80-day semester for all strategies, implemented at various screening frequencies and vaccination rates, in the base-case scenario, for 75% compliance ($\eta = \alpha = 75\%$) and 60% initial campus-wide vaccine coverage ($L_s = L_f = 60\%$).

		Infections					Deaths	Hospitalizations			
Strategy	Test Frequency	Peak number	Peak day	Student number	Faculty number	Total number	Total number	Peak number	Student number	Faculty number	Total number
**120 vaccines/day**											
	every 14d	9	25	347	3	350	0	1	1	1	2
	every 7d	7	25	277	3	280	0	1	1	1	2
$V - S_F$	every 3 d	4	23	168	2	170	0	1	0	1	1
	every 2d	3	16	129	1	130	0	1	0	1	1
	every 1d	2	16	70	0	70	0	1	0	1	1
	every 14d	11	25	376	4	380	0	1	1	1	2
	every 7d	10	25	337	3	340	0	1	1	1	2
$V - S_P$	every 3d	8	25	247	3	250	0	1	1	1	2
	every 2d	7	25	208	2	210	0	1	0	1	1
	every 1d	5	25	139	1	140	0	1	0	1	1
$V - \overline{S}$	N/A	12	25	446	4	450	0	1	1	1	2
**60 vaccines/day**											
	every 14d	11	30	910	10	920	1	4	5	3	8
	every 7d	8	24	703	7	710	0	3	4	3	7
$V - S_F$	every 3 d	5	24	386	4	390	0	2	3	3	6
	every 2d	3	23	268	2	270	0	2	2	3	5
	every 1d	2	16	138	2	140	0	2	1	3	4
	every 14d	12	30	1,008	12	1,020	1	4	6	3	9
	every 7d	10	31	871	9	880	0	4	5	3	8
$V - S_P$	every 3d	7	31	624	6	630	0	3	3	3	6
	every 2d	6	37	505	5	510	0	2	3	3	6
	every 1d	5	46	337	3	340	0	2	2	3	5
$V - \overline{S}$	N/A	15	26	1,185	15	1,200	1	5	7	3	10
**30 vaccines/day**											
	every 14d	13	31	1,304	16	1,320	2	6	16	7	23
	every 7d	10	31	1,069	11	1,080	2	5	13	7	20
$V - S_F$	every 3 d	5	24	635	5	640	2	3	8	7	15
	every 2d	3	23	437	3	440	2	3	6	7	13
	every 1d	2	16	218	2	220	1	2	4	7	11
	every 14d	14	31	1,382	18	1,400	2	6	17	7	24
	every 7d	11	31	1,227	13	1,240	2	5	14	7	21
$V - S_P$	every 3d	6	31	902	8	910	2	4	10	7	17
	every 2d	5	37	734	6	740	1	4	8	7	15
	every 1d	3	44	476	4	480	1	3	5	7	12
$V - \overline{S}$	N/A	17	30	1,537	23	1,560	2	7	19	8	27
**No vaccination**											
$\overline{V} - \overline{S}$	N/A	20	31	1,798	32	1,830	2	8	23	9	32
	every 14d	15	31	1,618	22	1,640	2	7	20	8	28
	every 7d	11	31	1,394	16	1,410	2	6	16	8	24
$\overline{V} - S_F$	every 3d	6	31	862	8	870	2	4	10	7	17
	every 2d	4	30	595	5	600	2	3	7	7	14
	every 1d	2	23	288	2	290	1	2	4	7	11

Table H: Total number of infections and peak number of hospitalizations over the 80-day semester for various strategies, implemented at various screening frequencies and a daily vaccination rate of 60 vaccines/day, in the base-case scenario, for 75% compliance ($\eta = \alpha = 75\%$) and different initial campus-wide vaccine coverage levels.

		$L_s : 30\%$, $L_f : 30\%$		$L_s : 60\%$, $L_f : 60\%$		$L_s : 90\%$, $L_f : 90\%$	
Strategy	Test Frequency	Total number of infections	Peak number of hospitalizations	Total number of infections	Peak number of hospitalizations	Total number of infections	Peak number of hospitalizations
**60 vaccines/day**							
$V - S_F$	every 14d	3,010	13	920	4	15	0
	every 7d	2,750	12	710	3	13	0
	every 3 d	1,820	8	390	2	10	0
	every 2d	1,190	5	270	2	8	0
	every 1d	490	3	140	2	5	0
$V - S_P$	every 14d	3,080	14	1,020	4	15	0
	every 7d	2,940	13	880	4	14	0
	every 3d	2,540	10	630	3	12	0
	every 2d	2,220	9	510	2	10	0
	every 1d	1,600	6	340	2	7	0
$V - \overline{S}$	N/A	3,180	15	1200	5	17	0

References

[1] Paltiel AD, Zheng A, Walensky RP. Assessment of SARS-CoV-2 screening strategies to permit the safe reopening of college campuses in the United States. JAMA Network Open. 2020;3(7):e2016818–e2016818.

[2] Poletti P, Tirani M, Cereda D, Trentini F, Guzzetta G, Sabatino G, et al. Association of age with likelihood of developing symptoms and critical disease among close contacts exposed to patients with confirmed SARS-CoV-2 infection in Italy. JAMA Network Open. 2021;4(3):e211085–e211085.

[3] Niche. William & Mary; Accessed on December 2021.

[4] COVID-Net. Laboratory-Confirmed COVID-19-Associated Hospitalizations; Accessed on November 2021.

[5] CDC. Demographic Trends of COVID-19 cases and deaths in the US reported to CDC; Accessed on November 2021.

[6] McChesney J, Bichsel J. The Aging of Tenure-Track Faculty in Higher Education: Implications for Succession and Diversity. College and University Professional Association for Human Resources. 2020;.

[7] Statista. Number of coronavirus disease 2019 (COVID-19) deaths in the U.S. as of November 24, 2021, by age*; Accessed on November 2021.

[8] Statista. Total number of cases of COVID-19 in the United States as of November 24, 2021, by age group; Accessed on November 2021.

[9] Statista. Number of COVID-19 vaccine doses administered in the United States as of November 17, 2021, by vaccine manufacturer; Accessed on November 2021.

[10] CDC. Pfizer-BioNTech COVID-19 Vaccine Overview and Safety (also known as COMIRNATY); Accessed on November 2021.

[11] CDC. Moderna COVID-19 Vaccine Overview and Safety; Accessed on November 2021.

[12] Centers for Disease Control. Different COVID-19 vaccines; Accessed on November 2021.

[13] CDC. CDC Updates and Shortens Recommended Isolation and Quarantine Period for General Population; Accessed on December 2021.

[14] Ito K, Piantham C, Nishiura H. Relative instantaneous reproduction number of Omicron SARS-CoV-2 variant with respect to the Delta variant in Denmark. Journal of medical virology. 2021;.

[15] Lyngse FP, Mortensen LH, Denwood MJ, Christiansen LE, Møller CH, Skov RL, et al. SARS-CoV-2 Omicron VOC Transmission in Danish Households. medRxiv. 2021;.
